# Extracellular Vesicles as Conveyors of Membrane-Derived Bioactive Lipids in Immune System

**DOI:** 10.3390/ijms19041227

**Published:** 2018-04-18

**Authors:** Krizia Sagini, Eva Costanzi, Carla Emiliani, Sandra Buratta, Lorena Urbanelli

**Affiliations:** 1 Department of Chemistry, Biology and Biotechnology, University of Perugia, Via del Giochetto, 06123 Perugia, Italy; krizia.sagini@studenti.unipg.it (K.S.); eva.costanzi@studenti.unipg.it (E.C.); carla.emiliani@unipg.it (C.E.); 2 Centro di Eccellenza sui Materiali Innovativi Nanostrutturati (CEMIN), University of Perugia, Via del Giochetto, 06123 Perugia, Italy

**Keywords:** extracellular vesicles, antigen presentation, prostaglandins, leukotrienes, specialized pro-resolving mediators, lysophospholipids

## Abstract

Over the last 20 years, extracellular vesicles (EVs) have been established as an additional way to transmit signals outside the cell. They are membrane-surrounded structures of nanometric size that can either originate from the membrane invagination of multivesicular bodies of the late endosomal compartment (exosomes) or bud from the plasma membrane (microvesicles). They contain proteins, lipids, and nucleic acids—namely miRNA, but also mRNA and lncRNA—which are derived from the parental cell, and have been retrieved in every fluid of the body. As carriers of antigens, either alone or in association with major histocompatibility complex (MHC) class II and class I molecules, their immunomodulatory properties have been extensively investigated. Moreover, recent studies have shown that EVs may carry and deliver membrane-derived bioactive lipids that play an important function in the immune system and related pathologies, such as prostaglandins, leukotrienes, specialized pro-resolving mediators, and lysophospholipids. EVs protect bioactive lipids from degradation and play a role in the transcellular synthesis of prostaglandins and leukotrienes. Here, we summarized the role of EVs in the regulation of immune response, specifically focusing our attention on the emerging role of EVs as carriers of bioactive lipids, which is important for immune system function.

## 1. Introduction

In multicellular organisms, cell-to-cell communication may take place through several mechanisms. Among these, a key role for extracellular vesicles (EVs) has been definitively established. EVs allow intercellular communication both at short and long ranges [1], and a consistent number of studies has provided evidence that EV-mediated intercellular communication is of fundamental importance for many functions in the immune system [2].

The term EV refers generally to vesicular structures surrounded by a lipid bilayer, which are released in the extracellular environment by eukaryotic cells. EVs can be classified by size and origin into three main categories: exosomes, microvesicles, and apoptotic bodies. Exosomes are characterized by their small dimensions (30–100 nm diameter) and round shape. They originate by the inward budding of late endosomal membranes, resulting in the progressive accumulation of intraluminal vesicles (ILVs) within endosomes, that become large multivesicular bodies (MVBs). After MVB fusion with the plasma membrane, vesicles are released into the extracellular space; for this reason, they have been termed “exosomes”. Microvesicles, which are also referred to as ectosomes, originate from plasma membrane outward budding. They are characterized by a wider size distribution (from 100 to 1000 nm) and a less regular morphology than exosomes. Finally, apoptotic bodies are released by dying cells during the later stages of apoptosis [3]. They have a larger size (500–5000 nm) than exosomes and microvesicles, although a few studies reported that apoptotic cells may also release smaller vesicles [4]. Despite the apparently simple classification of EVs based on their subcellular compartment of origin, from an operational point of view, it is difficult to obtain vesicles that come exclusively from one source, i.e., plasma membrane or MVBs. This is mainly because purification methods rely on the same biophysical properties (namely size); furthermore, commonly used experimental procedures, such as ultracentrifugation and ultrafiltration, can affect vesicle biophysical properties, prompting their aggregation, fusion, and co-precipitation with other molecules [5,6]. Exosomes and microvesicles are enriched with different proteins, that are therefore routinely used as markers [7]. Several studies have proposed Alix, Tsg101, CD63, and CD9 as exosomal markers. However, it has recently become clear that even if these molecules are enriched in exosomes, they are not exclusively associated with this type of vesicles; they can be also found in microvesicles [5,7].

The content of EVs has been extensively investigated. EVs carry, in their inner core or in their lipid bilayer, bioactive compounds such as proteins, lipids, nucleic acids, and metabolites. Most of these molecules are structural components, but they are also signalling mediators. The biochemical composition of EVs is not the same of the releasing cell. However, it is reminiscent of it, and of its pathophysiological condition. So, theoretically it could be possible to identify an EVs subpopulation deriving from a specific cell type, either in physiological or pathological condition, using specific biomarkers, although more studies in this direction are required [8].

EVs have been mostly characterized by their protein and nucleic acid content. In most proteomic studies, proteins linked to their endosomal origin have been identified in exosomes, namely proteins involved in MVBs biogenesis (e.g., Alix and Tsg101), proteins associated with lipid microdomains, such as integrins and tetraspanins (e.g., CD63, CD9, CD81 and CD82), transport and fusion proteins (e.g., GTPase, annexins, flotillins), heat shock proteins (HSP70, HP90) [6,9]. In addition to common polypeptides, exosomes may be enriched with cell-specific proteins. Of interest, several studies have found polypeptides in exosomes that directly or indirectly regulate the immune response. These components have been shown to include peptide-bound major histocompatibility complex (MHC) class I and II, T-cell stimulatory molecules (B7.2, ICAM-1), and other immunologically active molecules such as milk fat globule-EGF factor 8 (MFG-E8), Fas ligand (FasL), galectin-9, TGF-β, TNF-α, and Natural Killer Group 2D (NKG2D) ligand [2]. Although it is not possible to identify unique signatures of microvesicles versus exosomes [5], their protein content appears to be enriched with matrix metalloproteinases and cytoskeletal components, such as β-actin and α-actinin-4 [3]. 

An increasing number of studies have demonstrated that EVs carry nucleic acids, namely microRNAs (miRNA), messenger RNAs (mRNA) and long non-coding RNAs (lncRNA). As for proteins, the subset of miRNAs enriched in EVs is different than the subset of miRNAs expressed in the cell of origin. Several studies have demonstrated that miRNAs carried by EVs can reach recipient cells, where they can affect the expression of target genes [9]. Of great relevance, it has been demonstrated [10] that EVs not only carry mRNAs, but that these mRNAs can be also expressed in recipient cells following EVs capture, therefore reinforcing the evidence that EVs can represent a previously unknown and unique means of horizontal gene transfer. Several miRNAs that have a direct role in the immune system have been found in EVs [11]: EVs released by dendritic cells (DC) deliver miR-155 and miR-146a, which are known to enhance and reduce inflammatory response, respectively [12]. EV-associated miR-150, which is released by both B and T cell-derived EVs, modulates adaptive immune responses [13].

The lipid content of EVs has been poorly investigated with respect to protein and RNA content, but it is interesting to note that as for proteins and RNAs, the EV lipid composition is different from that of the releasing cell, although it is reminiscent of it. Generally, EVs are enriched in cholesterol and sphingolipids, indicating that their membrane composition resembles that of lipid rafts [14,15,16,17]. EVs carry mostly saturated fatty acids, but monounsaturated and polyunsaturated fatty acids are also present. Among the unsaturated species, arachidonic acid, the precursor of several lipid mediators, is one of the most represented [18].

## 2. The Role of Extracellular Vesicles (EVs) in the Regulation of Immune Response

EVs became of interest for immunologists in 1996, when Raposo et al. [19] demonstrated that B cells infected with the Epstein–Barr virus fuse their MVBs with the plasma membrane and release EVs of nanometric size outside of the cells. Afterwards, much evidence has been provided that EVs produced by both non-immune and immune cells have an important role in the regulation of intercellular communication during both adaptive and innate immune responses [20,21]. The well-demonstrated stimulatory and tolerogenic properties of EVs prompted their use as therapeutic tools. For example, they have been exploited either to break the tolerance against tumor antigens in anti-cancer immunotherapy, or as immunosuppressive agents in inflammation-driven immune pathologies, namely autoimmune diseases [22].

### 2.1. Adaptive Immune Response

EVs carry both MHC class II [19] and MHC class I [23] molecules, and this finding immediately indicated that EVs could play a role in adaptive immune response. Currently, much evidence has been provided that EVs from both non-immune and immune cells are involved in antigen presentation, both directly and indirectly. 

The best characterized process in which EVs are involved is the direct antigen presentation to T cells. MHC class I and class II complexes are present on the surface of EVs released by antigen presenting cells (APCs), and therefore can potentially directly stimulate CD8^+^ and CD4^+^ T cells, respectively. Professional APCs such as DCs release EVs in a constitutive manner, but their secretion is increased upon different types of stimulation, such as toll-like receptors (TLR) binding on DCs [24]. EVs released by DCs stimulated primed CD4^+^ T cells, but failed to stimulate naïve CD4^+^ T cells, which require bystander mature DCs [25,26]. The maturation of DCs is also a relevant issue in antigen presentation by DC-derived EVs, as several reports indicated that mature DC-derived EVs were more efficient in elicit immune responses than EVs from immature DCs, which instead promoted tolerance [27,28]. EVs from DCs also induced antigen-specific CD8^+^ T cell responses [29]. Besides, when DCs were pulsed with peptides from tumors, they released EVs that were able to prime specific CD8^+^ cells, i.e., cytotoxic T lymphocytes. In turn, these lymphocytes eradicated or suppressed the growth of established murine tumors [23]. These results have prompted the application of EV-based cell-free vaccines as an alternative to DC cellular therapy for suppressing tumor growth, leading to a phase II clinical trial [30,31]. Other APCs such as B cells also release EVs in a constitutive manner, and their secretion is increased following B-cell receptor (BCR) cross-linking [32]. EVs secreted by B cells could stimulate CD4^+^ T cell activation [33,34].

Besides direct antigen presentation by professional APCs to T cells, EVs released by any cell type can be captured by APCs and then present antigens in an indirect manner, thus eliciting either a stimulatory or inhibitory T cell response. As a matter of fact, once EVs released from a tissue or cell type are captured by DCs, the vesicular peptide–MHC complexes can be degraded, becoming a source of peptides for APCs that can directly interact with T cells [27,35,36]. The mechanism of indirect antigen presentation by EVs becomes very important when cells are infected, thus representing an important aspect of host–pathogen interaction [37]. Indeed, endothelial cells infected with cytomegalovirus (CMV) transport virus-derived peptides that activate CD4^+^ T cells against CMV [38], resulting in an anti-viral response. The mechanism is also a relevant feature of anti-tumor immune response. The presence of tumor antigen peptides associated with MHC complexes in EVs released from tumor tissue has been demonstrated to elicit specific cytotoxic T lymphocytes response [39]. 

EVs may deliver to APCs not only peptide–MHC complexes, but also whole antigens. Indeed, tumor antigens can be captured within EVs. Once released, these EVs can be captured, and their antigens can be processed by professional APCs such as DCs. This event allows the association of antigen-derived peptides with MHC on APCs, eliciting a specific immune response [40]. 

T cells are not only targets of EVs released by APCs; they can also release their own EVs. T cell EVs have been shown to play many roles, both in adaptive and innate immune responses. Although they are released constitutively, their secretion can be increased by T cell receptor (TCR) triggering [41]. T cell-derived EVs promote immunogenicity by inducing T cell proliferation [42], as well as through the regulation of gene expression in APCs [43].

Despite the initial enthusiasm of early findings demonstrating the immunogenic potential of EVs, more recent investigations provided evidence that the promotion of tolerogenesis by EVs is as important as the elicitation of an immune response [21], complicating the picture. Indeed, EVs not only induce an immune response against tumor antigens, they also promote the function of T regulatory cells (Tregs), which are key players in the regulation of self-tolerance. In addition, they inhibit the activity of natural killer (NK) cells, which are crucial components of the innate immune system that do not require pre-stimulation to perform their effector functions [44]. Finally, EVs from tumors can induce lymphocytes and T cell death by apoptosis via FasL [45,46]. When T cells are activated, they also release EVs bearing FasL, which target bystander T cells, inducing apoptosis [47]. A few studies also reported an immunosuppressive function for EVs isolated from body fluids such as milk [33] and bronchoalveolar lavage fluid (BALF) [48].

### 2.2. Innate Immune Response

Innate immune system cells release EVs that act as paracrine mediators, inducing or propagating inflammatory signals during infections and inflammatory diseases, including autoimmune disorders. However, in addition to promoting inflammation, EVs released by innate immune cells can also contribute to a negative regulation of inflammation, for example by delivering endogenous pro-resolving lipid mediators. At the beginning, the involvement of EVs in innate immunity modulation was suggested by the discovery that EVs carry several cytokines, namely multiple TNF superfamily members, TGF-β, IL1α, and IL1β. In addition, other components affecting the innate immune response, such as TLRs and pathogen-associated molecular patterns (PAMPs), were also found to be associated with EVs [49,50].

Professional APCs such as DCs and macrophages release EVs containing IL1β, which is a major driver of the innate immune response, through a non-classical secretion pathway [51]. EVs from both immature and mature DCs also contain multiple TNF superfamily ligands such as TNF, FasL, and Trail, which directly bind to surface receptors on NK cells to enhance their cytotoxic activity [52,53]. DC-derived vesicles have also been shown to induce NF-κB activation in microglia cells, which may play a role in the inflammatory response that is observed in the CNS during experimental autoimmune encephalomyelitis [54].

Beyond their relevance in the antigen presentation introduced in Section 2.1, EVs isolated from macrophages were also shown to play additional roles, as they have been implicated in inflammation-induced programmed cell death and the differentiation of naïve monocytes into macrophages [51,55,56]. The role of macrophage EVs is also relevant in infectious diseases, as EVs have been demonstrated to carry pathogenic antigens. When macrophages are infected with several species of *Mycobacteria* [50,57], they incorporate mycobacterial components. Beyond developing an antigen-specific response, these EVs when incubated with naïve macrophage enhance the release of proinflammatory cytokines and chemokines, and promote the recruitment of other immune cells, thus prompting granuloma formation [58,59]. Similar results were obtained when macrophages were infected with parasites [60]. 

Macrophage EVs have also been involved in the activation of inflammatory responses associated with vascular inflammation and atherosclerosis. Specifically, it has been shown that EVs released by macrophages promote leukocyte migration by the upregulation of intracellular adhesion molecule (ICAM-1) [61], participating in the regulatory network that prompts wall infiltration. Besides, macrophage EVs have been shown to affect endothelial cell (EC) function by regulating integrin trafficking [62]. The release of EVs from other immune cells has also been demonstrated to affect the function of ECs. In the case of EVs released by neutrophilic granulocytes, a pro-inflammatory role towards ECs was indicated by the evidence that these vesicles stimulate the EC secretion of the pro-inflammatory cytokine IL6 and induce myeloperoxidase-mediated EC damage [63,64]. However, the interactions between immune cells and vascular cells is complex, and there is evidence that EVs from different blood sources may have different actions on ECs, enhancing or inhibiting inflammation [65]. 

The inflammatory role of EVs released by neutrophilic granulocytes was confirmed by the finding that they possess an antibacterial effect that is selective for specific bacterial strains [66]. However, several studies also provided evidence of an anti-inflammatory effect of these EVs through different mechanisms [67,68]. Neutrophil-derived EVs were reported to increase the secretion of the anti-inflammatory cytokine TGF-β1 from monocytes, thus interfering with the maturation of monocyte-derived DCs [69]. They were also reported to prompt the release of lipid mediators, stimulating the phagocytosis of dying cells by macrophages [70]. In addition to neutrophilic granulocytes, other granulocytes, such as mast cells, release EVs with immunomodulation properties, as mast cell EVs were demonstrated to induce the maturation of DCs and the activation of and B and T lymphocytes [71,72].

EVs released by NK cells have raised considerable interest in the oncology field, as they have been shown to exhibit cytotoxic activity against tumor cells and activate immune cells [73,74]. Moreover, NK cells are the target of EVs that are released by several cell types. These EVs are able to activate NK cells, conferring on them the ability to recognize tumor cells and reduce their growth [75,76]. On the other hand, EVs containing NKG2D receptor ligands were shown to downregulate NK function and reduce NK cytotoxicity, thus favouring tumor escape [77,78].

## 3. Lipids as Signaling Mediators in the Immune System

Lipid mediators play a pivotal role in immune signaling and inflammatory processes. Indeed, defects in the metabolism of lipid mediators or in their receptors account for several inflammatory and immune disorders [79]. In this section, we summarize the most relevant features of lipid mediators and their involvement in immune system signaling, in order to introduce key findings about the role of EVs as conveyors of membrane-derived bioactive lipids in the next section.

Based on their biosynthetic origin, lipid mediators can be grouped into two different classes, i.e., polyunsaturated fatty acids (PUFA)-derived mediators and lysophospholipids, as extensively reviewed elsewhere [80]. Bioactive lipids derived from PUFA can be further divided into two subclasses: lipid mediators deriving from the ϖ6 arachidonic acid (AA, 20:4 n6), which include thromboxanes (TGs), prostaglandins (PGs), leukotrienes (LTs), and lipoxins (LXs), and lipid mediators deriving from ϖ3-PUFA, i.e., E-series resolvins and D-series resolvins, protectins, and maresins. Except for LXs, lipids mediators derived from ϖ6-PUFA (generally known as eicosanoids) are pro-inflammatory, whereas all the mediators derived from ϖ3-PUFA promote the resolution of inflammation. It is noteworthy that another class of lipid mediators, i.e., endocannabinoids (eCBs), also originates from PUFA metabolism, but their classification is less clear, as eCBs may derive from either ϖ6- or ϖ3-PUFA [80,81]. Membrane-derived bioactive lipids derived from lysophospholipids (LPLs) can be divided into lysoglycerophospholipids (LGPLs), which are characterized by glycerol as the backbone, and lysosphingophospholipids (LSLs), which are characterized by sphingosine as the backbone [82].

### 3.1. Classical Eicosanoids

AA-derived eicosanoids exert a pivotal role in immune response. Cells of myeloid lineage, such as platelets, monocytes, macrophages, neutrophils, and mast cells are major producers of eicosanoids, although, except for LTs, they are also synthesized by a variety of non-immune cell types [83]. Free AA can be metabolized by three different classes of enzyme: lipoxygenases (LOX), which produce LTs, hydroxyeicosatetraenoids (HETEs), and LXs; cyclooxygenases (COX), which produce PGs, prostacyclins, and TXs; and P450 (CYP) epoxygenases, which produce HETEs [84,85].

LTs are considered potent inflammatory mediators. Their synthesis begins with the oxygenation of AA by 5-LOX to form a precursor, which is further dehydrated to LTA4 in multiple cells, including leucocytes and macrophages. LTA4 is converted into LTB4 [86], or alternatively, it can be conjugated to glutathione (GSH) by LTC4 synthase to form a cysteinyl-leukotriene (Cys-LT), LTC4 [87]. Other Cys-LTs are formed by the hydrolytic removal of γ-Glu and Gly from the LTC4 glutathione moiety (yielding LTD4 and LTE4) [88]. LTs bind to G-protein-coupled receptors (GPCRs), and specifically cysteinyl-LT mediators bind to CysLT1 and CysLT2 receptors, whereas LTB4 binds to BLT1 and BLT2 receptors [89]. LTs’ action on inflammatory response depends on the type of LTs produced, and on the type of receptor expressed by the target cell. LTs function as chemotactic molecules that can facilitate the recruitment and accumulation of leukocytes at inflammatory sites [90]. Besides, Cys-LTs play a pivotal role in respiratory inflammation, as they regulate airway remodelling, mucus secretion, and bronchoconstriction. For this reason, they are the target of anti-asthmatic and anti-allergic therapies [91]. 

LTs, like other eicosanoids such as PGs and LXs, feature the peculiarity of a biosynthesis that has been defined as “transcellular”. In fact, although some cells express all the enzymes that are necessary to produce biologically active LTs, it has been shown that the biosynthesis of LTs is often the result of a cell-to-cell interaction involving the transfer of biosynthetic intermediates. In more detail, the transcellular synthesis requires both a donor cell to synthesize and release one component of the biosynthetic cascade, and an accessory cell to take up that intermediate and process it into the final biologically active product. For example, the LTA4 that is synthesised in neutrophils, which express the 5-LOX enzyme, is released and taken up by cells, such as platelets, lacking 5-LOX but possessing LTA4 hydrolase, and for this reason, they are able to produce LTB4 from LTA4 [92].

PGs belong to prostanoids, which also include prostacyclins and TXs. These molecules derive from AA through the action of COX enzymes that generate PGH2, which is a common substrate for a series of specific enzymes producing the various members of prostanoid subclasses [93]. There are two different COX isoforms: COX-1 is a constitutively expressed enzyme, whereas COX-2 is upregulated in inflammatory conditions. PGH2 is an instable metabolite that is quickly converted into the biologically active molecules downstream, such as PGD2, PGE2, and PGF2α. It is also the precursor for the synthesis of prostacyclin PGI2 and TXs by CYP family enzymes [94]. Prostanoids exert their biological effects by binding to GPCRs, i.e., EP1 to EP4 for PGE, DP1 and DP2 for PGD, and PGI and TX receptors for prostacyclins and TX, respectively [95]. Several studies have provided evidence that demonstrates that prostanoids can also bind to nuclear receptors of the PPAR (peroxisome proliferator-activated receptor) family [96].

As for LTs, the differential expression of prostanoid biosynthetic enzymes in cells that are present at the inflammation site determines the type and the level of the prostanoid synthesized, whereas the differential expression of the receptors on the target cells determines the type of signal that is activated. The sum of these factors is integrated in a complex picture to give a pro-inflammatory or anti-inflammatory outcome to the prostanoid action. Perhaps the most relevant feature related to the pro-inflammatory or anti-inflammatory action of prostanoids is the evidence that PGs are also essential intermediates for the synthesis of LXs, resolvins, and protectins, which are lipid mediators that are specifically involved in the resolution of inflammation [97].

LXs are characterized by anti-inflammatory and pro-resolving activities, thus representing an exception to the common idea that AA-derived mediators have only pro-inflammatory actions. The main LXs that are produced endogenously in mammals are LXA4 and LXB4, but more recent studies have identified two additional members of the family [98]. LXs are generated by cooperation between 5-LOX and 12-LOX or 15-LOX through different routes involving the transcellular interaction between various cell types, such as neutrophils, platelets and tissue resident cells. LXA4 binds with high affinity to the GPCR formyl peptide receptor 2 (ALX/FPR2), whereas the receptor for LXB4 remain unclear [99]. Myeloid cells represent the major target for LXA4 and are responsible of many physiological responses that are associated with LXs, including the inhibition of LTs synthesis [100] and the attenuation of NF-κB pro-inflammatory responses [101]. In addition, these lipid mediators participate in the resolution of inflammation by stimulating the phagocytosis of apoptotic neutrophils and downregulating neutrophil responses [102].

### 3.2. Endocannabinoids (eCBs)

The eCB family comprises a large group of PUFA derivatives that are produced by most tissues and immune cells. The first and the most intensively studied eCB are ϖ6-derived *N*-arachidonoylethanolamide (AEA or anandamide, an amide of AA and ethanolamine), and 2-arachidonoylglycerol (2-AG, an ester of AA and glycerol). It has been reported that these mediators may also originate from ω3 fatty acids, as ethanolamides of docosahexaenoic acid (DHA) and eicosapentaenoic acid (EPA) [103]. Among lipid mediators, eCBs are considered the most potent immunoregulatory molecules; they are capable of modulating both stimulatory and inhibitory signaling. AEA is considered mostly anti-inflammatory, whereas 2-AG has displayed both pro-inflammatory and anti-inflammatory properties [104]. Regardless of their biosynthetic origin, all eCBs are agonists of CB1 and CB2 receptors. These are GPCRs usually coupled to heterotrimeric G_i_ alpha subunits [105].

### 3.3. Specialized Pro-Resolving Mediators (SPMs)

Specialized pro-resolving mediators (SPMs) include LXs (see above), resolvins (Rv), protectins (PD), and maresins (MaR). These molecules all exert potent anti-inflammatory and pro-resolving actions within the picogram to nanogram range [106]. SPMs arrest leukocytes infiltration and enhance both bacterial phagocytosis by neutrophils and macrophage-mediated phagocytosis of neutrophils and cell debris. As they do not appear to compromise the host immune competence, they have raised considerable interest for the treatment of many conditions characterized by an excess of inflammation [107].

Resolvins (resolution phase interaction products) include two subfamilies, each consisting of several members: the E-series (RvE1 and RvE2) and the D-series (RvD1 to RvD6), which are produced enzymatically from EPA and DHA, respectively. RvE1 is synthesized by acetylated COX-2 or via a cytochrome P450 pathway. Indeed, in the presence of aspirin, COX-2 can be acetylated, and the acetylated COX-2 does not generate PGs anymore but is still able to convert EPA into an E-series resolvin precursor [108]. RvE1 binds to two GPCRs, i.e., ChemR23 and BLT1 [109]. RvE1’s anti-inflammatory and pro-resolving activities are mostly due to its action on granulocytes, as RvE1 inhibits the migration of granulocytes across the endothelium and promotes their removal by stimulating macrophages’ phagocytosis of apoptotic granulocytes [110]. RvD1 and RvD2 are synthesized via DHA lipoxygenation by 15-LOX, then the intermediate can be converted into several RvDs [105]. RvD1 binds to ALX/FPR2 and GPR32 receptors, which are expressed predominantly on immune cells [111]. Similar to other lipid pro-resolving agents, D-series Rv possesses the ability to inhibit the migration of granulocytes and attenuate their activation. Besides, these molecules also increase the phagocytic ability of macrophages, promoting bacterial clearance [112,113].

Protectins (PD) are also formed via DHA lipoxygenation, in brain and human peripheral blood. Two members of this class have been identified, PD1 (which is named neuroprotection D1 when produced by neural tissue) and PDX [105]. PD1 exerts anti-inflammatory actions, i.e., it reduces granulocyte numbers in vivo, and decreases the production of inflammatory cytokines by glial cells [114]. PDX, an isomer of PD1, is not only involved in the inflammation resolution processes, it also exerts anti-aggregatory properties in human platelets [115].

Maresins (Macrophage Mediators in Resolving Inflammation and Organ Regeneration, MaR) have been the last family of resolving lipid mediators to be discovered. They include two members, MaR1 and MaR2 [116], which are produced in macrophages by 12-LOX. MaRs have shown resolving properties comparable to those of other resolving mediators, i.e., they attenuate neutrophil recruitment and stimulate macrophage phagocytic ability. Moreover, MaR1 showed inhibitory properties towards the NF-κB pathway [117].

### 3.4. Lysophospholipids

Lysophospholipids originate from membrane phospholipids via the enzymatic removal of an ester-bound fatty acid by phospholipases A or sphingomyelinases. Among lysoglycerophospholipids, lysophosphatidic acid (LPA) and lysophosphatidylchline (LPC) are the most active molecules. Extracellularly, LPC is produced from phosphatidylcholine (PC), and circulates in association with albumin or other lipoproteins, whereas LPA is produced via removal of the LPC choline moiety by a lysophospholipase D termed autotaxin (ATX) [118]. Extracellular LPA binds to specific cell-surface GPCRs, which are named from LPA1 to LPA6 [119]. LPC has been considered for a long time to be only a precursor of LPA, but this view changed when two GPCRs for LPC were identified, i.e., GPR4 and G2A, demonstrating that LPC also acts as an independent lipid mediator [120]. Despite the expression of several LPA receptors in immune cells, the role of LPA in immune response and inflammation is not well understood. LPA has been shown to enhance the motility of T cells in vitro [121], and inhibit CD8 T cell activation and proliferation [122]. It stimulates multiple pro-inflammatory activities, including the degranulation of neutrophils and the migration of eosinophils and immature DCs [123]. Besides, in vivo ATX expression is induced at the site of inflammation, leading to the local production of LPA. For this reason, it has been proposed that the ATX–LPA axis may regulate lymphocyte extravasation in lymph nodes [124]. LPC acts on the immune system via the G2A receptor, and modulates several cellular responses, namely macrophages and T cell migration [125,126], neutrophils activation [127], and the phagocytic clearance of apoptotic cells and activated neutrophils [128].

The main active metabolite among LSLs is sfingosine-1-phosphate (S1P). It derives from ceramide, which is converted by ceramidase into sphingosine and then phosphorylated into S1P by sphingosine kinases. After being exported extracellularly, S1P acts as a soluble lipid mediator that interacts with five GPCRs termed S1P1 to S1P5 [129]. S1P regulates lymphocyte trafficking by serving as a chemotactic ligand that controls B lymphocytes egress from lymphoid tissues [130]. The inhibition of S1P signaling causes lymphopenia and the sequestration of T cells in lymphoid organs [131]. Besides, S1P signaling regulates the trafficking of other types of immune cells, such as DCs, mast cells, monocytes, macrophages, and neutrophils [132].

## 4. EVs as Conveyors of Membrane-Derived Bioactive Lipids in Immune System

Currently, several studies have provided evidence that EVs not only contain lipids with a structural function, i.e., lipids belonging to the double layer membrane surrounding the vesicles, but also function as conveyors of membrane-derived bioactive lipids [17]. Lipids are poor water-soluble molecules by definition, so to reach their targets, they need to either act at short range or be transported to distant sites by specific carriers, such as lipoproteins. In addition, the transcellular biosynthesis implicates a cell-to-cell transportation route to complete the synthesis of several lipid mediators, namely PGs and LTs. In this context, EVs have gained attention as an alternative transportation route that is able to protect lipid mediators and/or their precursors from degradation and is also capable of acting either at short range and/or being transported by circulating fluids at distant districts of the body. An implication of this finding is that the regulation of EVs release is in turn an additional manner to modulate lipid-mediated cell signaling to neighbour tissues.

Evidence that PGs are present in EVs has been initially provided by a study on T cells that demonstrated that vesicular PGE2 is enriched in T cell derived-EVs, compared with EVs isolated from the supernatants of cultured tumor cells. The authors demonstrated that tumor progression promoted by myeloid-derived-suppressor cells (MDSC) was dependent on vesicular PGE2 [133] (Table 1). Then, it was reported that EVs from RBL-2H3 mast cells carry not only free fatty acids such as AA, which is the precursor of eicosanoids, but also PGs such as PGE2 and 15d-PDJ2 [134]. The same group also found that these vesicles contain enzymes involved in the biosynthesis of PGs (COX1 and COX2), indicating that the function of EVs may be not limited to the transport of PGs, but they could represent a production site for these lipid mediators. Further evidence that EVs may transport PGs and interact with immune cells came from recent work by Deng [135,136]. The authors demonstrated that intestinal mucosa-derived exosome-like vesicles contain PGE2 and can migrate to the liver, where they activate the Wnt pathway to induce NK cells’ anergy. These findings indicated that the PGE2 carried by EVs acts as an endogenous immune modulator between the liver and intestine to maintain liver NK cells’ homeostasis.

PGs are not the only AA-derived lipid mediators that have been localized in EVs. Esser et al. [137] showed that EVs from macrophages and DCs contain functional enzymes for the biosynthesis of LTs. Specifically, they reported that EVs released by macrophages and DCs carry LTC4. However, in macrophages and DCs LTB4 and LTC4 are the major products of LT biosynthesis, respectively. The finding suggested that cells and EVs possess different set of enzymes, implicating the vesicular formation of LTC4. LTA4 hydrolase and LTC4 synthase were also localized in EVs isolated from human plasma [137]. Further evidence that vesicular LTs could have physiological effects came from the work of Majumdar et al. [138]. In this case, the authors demonstrated that in neutrophils, LTB4 and its synthesizing enzymes are localized in intracellular MVBs, which are the late endosomal compartment where exosomes are synthesized. When vesicles are released upon fusion of the MVBs with the plasma membrane, they activate resting neutrophils and elicit chemotactic activity in a LTB4 receptor-dependent manner.

LTs carried by epithelial cell EVs were also found to have an immunoregulatory function and are involved in immune-related pathologies. Lukic et al. [139] investigated LT cross-talk between myeloid cells and pulmonary epithelial cell-derived EVs, demonstrating that EVs from human lung carcinoma cell line contained γ-glutamyltransferase 1, the enzyme that converts LTC4 in LTD4, and were able to increase LTD4 formation. LTD4 is a CysLTs that elicits mucus secretion and smooth muscle contraction, resulting in bronchoconstriction and vasoconstriction, and thus contributing to symptoms in lung inflammatory diseases, such as asthma. Further evidence of the involvement of EVs in allergies was obtained by investigating EVs that were isolated from bronchoalveolar lavage fluid (BALF). Proteome analysis of this material confirmed the presence of LTA4 hydrolase, which converts LTA4 into LTB4. In addition, its level was upregulated in patients with sarcoidosis and mild allergic asthma to birch pollen [140,141]. These findings clearly indicated that BALF vesicles might contribute to subclinical inflammation by generating LTC4 in the airway epithelium, and their release might represent an important mechanism of tissue homeostasis.

A more complex picture of the lipid mediators that are involved in inflammation and are present in EVs has been drawn by the Serhan group. Through the systematic lipidomic profiling of EVs that were isolated from inflammatory exudates during the time course of a self-limited inflammation, the authors succeeded in identifying the precursors for specialized pro-resolving mediators (SPMs) [142]. Specifically, they found in EVs hydroxyDHA (HDHA), namely 14-HDHA, which is a precursor of MaR, and 17-HDHA, which is the precursor of D-series Rv. The level of these precursors was high during the initial phase of acute inflammatory response, decreased during the peak of inflammation, and accumulated in resolution. Based on these results, the authors constructed novel nanoparticles (NPs) containing RvD1 or LXA4 analogues and demonstrated that these biomimetic NPs reduced the influx of granulocytes and shortened resolution intervals in a mouse model of peritonitis [142]. Furthermore, when secretory PLA2 was added to resolving EVs, it liberated esterified precursors from EVs, suggesting that soluble phospholipase could act directly on EVs. In another study, Dalli et al. [70] demonstrated that EVs from activated neutrophils prompt the removal of apoptotic or necrotic cells by phagocytic cells via the stimulation of SPMs production. However, in this study, the authors failed to detect mature SPMs directly in EVs.

eCBs are another class of lipid mediators whose presence in EVs has been reported. The endocannabinoid system is involved in immunoregulation and neuroprotection, and despite a different hypothesis proposed to explain the transport of eCBs among cells, the matter is still under debate. In 2015, Gabrielli et al. [143] reported that an active eCB, i.e., anandamide, was present both in large and small EVs that were released by microglial cells. These vesicles could activate CB1 receptor and inhibit presynaptic transmission in target neurons, thus indicating that the EV transport of eCBs has a specific signaling function.

The presence of biologically active lipids derived from glycerophospholipids, namely LGPL, in EVs has also been reported, but their relative amount was dependent on the cell model. In EVs from fibroblasts, LPC and LPA were the most abundant species [17], as well as in EVs released by platelets [144], whereas in EVs released by colorectal cancer cells, LPE and LPS were more abundant than LPC [16]. As they represent the structural components of the vesicle double layer membrane, this early finding obtained by lipidomic studies initially did not attract much interest. However, a recent study demonstrated that autotaxin, the secreted lysophospholipase D that hydrolyses LPC to produce LPA, binds to the surface of EVs outside the cell. In this way, vesicular LPC can be converted into LPA, which can be locally released to activate the LPA receptors on the surface of the target cells [145]. Autotaxin has also been detected in the EVs that were released from fibroblasts, with a certain prevalence in the EVs released by cells undergoing senescence [17], which are known to be cleared by immune system cells to maintain tissue homeostasis.

The association of LSLs with EVs has been reported by Deng et al. [146]. This study showed that enterobacteria-secreted particles induce the production of exosome-like S1P-containing particles by intestinal epithelium, and that these EVs drive tumorigenesis in a Th17-dependent manner, possibly interfering with the recruitment of neutrophils and macrophages to altered and/or inhibiting Tregs. Overall, these findings provided evidence that the LPLs associated with EV do not only possess a structural role (in view of their conical shape to keep the right curvature of the EVs’ membrane), they also have a role in LPLs’ transport and signaling.

## 5. Conclusions

Compelling evidence shows that both large and small EVs do not only contain structural lipids, they may also act as lipid conveyors of bioactive lipids, transporting them to target cells and protecting them from degradation. This finding explains the previous observation that several lipid mediators, such as for example LPA, are present in circulating blood; at the same time, due to their relative hydrophobicity, they cannot just circulate as soluble molecules, and need a specific carrier. It is already known that lipoproteins may function as carriers, but EVs have also demonstrated that they play a role in this context. In the case of lipid mediators that are derived from PUFA, EVs also represent an additional manner through which substrates and enzymes can be exchanged between cells, indicating that they could have a very important function in the “transcellular” synthesis of leukotrienes and prostaglandins. Finally, a few lipid mediators transported by EVs, such as leukotrienes, have an important action as chemotactic agent, and contribute to the pathogenesis of diseases such as asthma. For this reason, the construction of biomimetic nanoparticles carrying pro-resolving lipid mediators could be important for future pharmacological approaches based on the modulation of lipid-mediated cell signaling, namely in pathological conditions due to exacerbated or chronic inflammation.

## Figures and Tables

**Table 1:** Summary of the studies reporting not only the presence but also a biological role for lipid mediators carried by extracellular vesicles (EVs). DC: dendritic cells; MAR: maresins; LPA: lysophosphatidic acid, S1P: sphingosine 1-phosphate.

Lipid Mediator	EVs Source	Biological Activity	Reference
*Prostaglandins*			
PGE2	T cells	Promotion of tumor progression	[130]
PGE2	Intestinal mucosa	Induction of NK cells anergy in the liver	[132,133]
*Leukotrienes*			
LTC4	Macrophages and DCs	Promotion of granulocytes migration	[134]
LTB4	Neutrophils	Neutrophil activation and elicitation of chemotactic activity	[135]
*Specialized Proresolving Mediators*			
MaR precursor, D-Rv precursor	Inflammatory exudates	Accumulation during inflammation resolution	[139]
*Endocannabinoids*			
Anandamide	Microglial cells	CB1 receptor activation and inhibition of pre-synaptic transmission	[140]
*Lysophospholipids*			
LPA	Hek cells	LPA receptors activation	[142]
S1P	Intestinal epithelium	Regulation of Th17 activity	[143]
